# Clusters of pain trajectories among patients with sickle cell disease hospitalized for vaso‐occlusive crisis: A data‐driven approach

**DOI:** 10.1002/jha2.114

**Published:** 2020-10-22

**Authors:** Angie Mae Rodday, Kimberly S. Esham, Nicole Savidge, Susan K. Parsons

**Affiliations:** ^1^ The Institute for Clinical Research and Health Policy Studies Tufts Medical Center Boston Massachusetts; ^2^ Division of Hematology/Oncology Tufts Medical Center Boston Massachusetts

**Keywords:** methodology, sickle cell disease, statistics

## Abstract

**Background:**

Vaso‐occlusive crises (VOCs) are the hallmark of sickle cell disease (SCD), with higher severity among hospitalized patients. Clustering hospitalizations with similar pain trajectories could identify vulnerable patient subgroups. Aims were to (a) identify clusters of hospitalizations based on pain trajectories; (b) identify factors associated with these clusters; and (c) determine the association between these clusters and 30‐day readmissions.

**Methods:**

We retrospectively included 350 VOC hospitalizations from 2013 to 2016 among 59 patients. Finite mixture modeling identified clusters of hospitalizations from intercepts and slopes of pain trajectories during the hospitalization. Generalized estimating equations for multinomial and logistic models were used to identify factors associated with clusters of hospitalizations and 30‐day readmissions, respectively, while accounting for multiple hospitalizations per patient.

**Results:**

Three clusters of hospitalizations based on pain trajectories were identified: slow (n = 99), moderate (n = 207), and rapid (n = 44) decrease in pain scores. In multivariable analysis, SCD complications, female gender, and affective disorders were associated with clusters with slow or moderate decrease in pain scores (compared to rapid decrease). Although univariate analysis found that the cluster with moderate decrease in pain scores was associated with lower odds of 30‐day readmissions compared to the cluster with slow decrease, it was nonsignificant in multivariable analysis. SCD complications were associated with higher odds of 30‐day readmissions and older age was associated with lower odds of 30‐day readmissions.

**Conclusions:**

Our results highlight variability in pain trajectories among patients with SCD experiencing VOC and provide a novel approach for identifying subgroups of patients that could benefit from more intensive follow‐up.

## 1 INTRODUCTION

Sickle cell disease (SCD), which is the most common inherited blood disorder in the United States, primarily affects people of African descent and Hispanics of Caribbean ancestry [1]. The hallmark of SCD is acute, painful vaso‐occlusive crisis (VOC), which can be frequent and difficult to treat, and result in high healthcare utilization [2]. Many patients attempt to manage their VOC at home, potentially resulting in underestimation of the pervasiveness of VOC [3, 4]. On the other hand, patients seeking medical care for VOC may represent more severe cases [5].

When patients with VOC present acutely to healthcare providers, they have usually exhausted home care options and require more aggressive pain management, typically delivered inpatient [6]. Prior studies described trends in pain scores during VOC hospitalizations, observing that pain scores decrease as the patient approaches discharge, sometimes reaching a plateau without complete resolution [7, 8, 9]. These studies primarily plotted or reported individual or mean pain scores for each hospitalization day. Although these methods produce visualizations of pain scores, they do not allow grouping or clustering of hospitalizations with similar pain trajectories, which could identify subgroups of vulnerable patients. In addition, the pain experience during a hospitalization may predict future hospitalizations and readmissions, further illustrating the potential use of grouping pain trajectories [9].

Growth mixture modeling and latent class growth analysis provides data‐driven approaches for identifying unobserved (“latent”) growth curves or trajectories, based on variables (eg, pain scores) collected over time [10]. However, using these methods for creating pain trajectories during a VOC hospitalization is challenging when length of stay (LOS) varies and missing data due to shorter LOS are not missing at random [10, 11]. Recent studies have introduced methods to create pain trajectories during hospitalizations, but have focused on portions of the hospitalization (eg, first 48 h, 3 days leading up to discharge) rather than the full hospitalization [12, 13]. Building on these approaches, we propose estimating the intercept and slope of pain score trajectories for each hospitalization and then using finite mixture modeling to identify clusters of hospitalizations based on these intercepts and slopes [14].

The overall goal of our study was to use our proposed methods to identify clusters of hospitalizations with similar pain trajectories among patients with SCD hospitalized for VOC. We then assessed whether patient (eg, gender) and clinical characteristics (eg, affective disorder, SCD complications, use of hydroxyurea, and genotype) were associated with these clusters. Based on prior research, we hypothesized that hospitalizations among patients with more severe SCD, patients with affective disorders, and patients prescribed hydroxyurea would have worse pain trajectories [15, 16, 17, 18, 19, 20]. Finally, we tested the association between clusters of pain trajectories and 30‐day readmissions, with and without adjustment for patient and clinical characteristics. We hypothesized that worse pain trajectories would be associated with 30‐day readmissions [9, 21, 22].

## 2 MATERIALS AND METHODS

### 2.1 Sample

**FIGURE 1:**
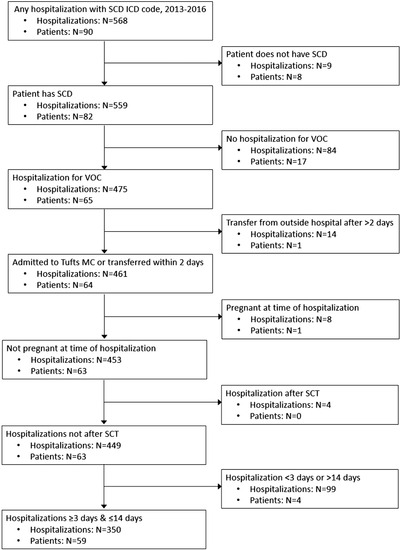
Development of cohort at patient and hospitalization level

We created a retrospective cohort of consecutive hospitalizations for VOC among adult patients (≥18 years) with SCD from 2013 to 2016 at Tufts Medical Center (Tufts MC), an academic medical center in Boston, MA. Hospital billing data were used to identify hospitalizations with ICD‐9 (282.6) or ICD‐10 (D57) codes for SCD. Trained study staff then reviewed the electronic medical record (EMR) to determine eligibility for each patient and hospitalization (Figure 1). All SCD genotypes (eg, hemoglobin [Hb] SS, Hb SC, Hb Sβ^+^ thalassemia, and Hb Sβ^0^ thalassemia) were included. Only hospitalizations for VOC were included, which was determined by review of the EMR for documentation of VOC or pain crisis. VOC could be due to SCD complications (eg, acute chest syndrome), but hospitalizations for SCD complications without a documented VOC were not included. Hospitalizations for procedures (eg, cholecystectomy) were excluded, as were hospitalizations during pregnancy or after stem cell transplant (SCT) where the pain experience and treatment may differ [23, 24]. We excluded transfer hospitalizations if patients spent >2 days at an outside hospital, as this would limit information about the early pain crisis. Hospitalizations <3 days were excluded because there were too few pain scores to create trajectories; hospitalizations >14 days were also excluded as their pain experience was hypothesized to be different (see Table A1 for comparison of hospitalization characteristics based on LOS exclusions). Exclusion of hospitalizations >14 days was based on statistical criteria (sparse data) and existing ranges of cutoffs used to define prolonged hospitalizations [7, 25, 26]. This study was approved by the Tufts Health Sciences Institutional Review Board.

### 2.2 Data

The study database had three levels of information: patient, hospitalization, and hospital day. Hospital days were nested within hospitalizations, which were nested within patient. Data were obtained from the hospital billing data or were abstracted from the EMR by trained study staff. Patient‐level data included date of birth, gender, race/ethnicity, and SCD genotype (Hb SS, Hb SC, and other).

Hospitalization‐level data included patient characteristics at the time of admission and characteristics or events occurring during the hospitalization. This included admission and discharge date, discharge status, insurance, whether the patient received routine care for SCD (“established”) at Tufts MC, history of SCD complications, documented affective disorder from the EMR (including depression or bipolar depression, as well as anxiety or prescription for selective serotonin reuptake inhibitor) in the prior 12 months, street address for calculating the Area Deprivation Index (ADI) [27], and home medications. LOS was calculated as discharge date minus admission date plus 1. Patients were defined as established at Tufts MC if they had a hematology clinic visit in the prior 12 months, or the EMR documented that they were established. A list of 13 SCD complications (see Appendix) was developed, based on known SCD complications and those included in the Adult Sickle Cell Quality of Life Measurement Information System (ASCQ‐Me) SCD Medical History Checklist [17]. SCD complications documented in the EMR in the 12 months prior to hospitalization were included. The ADI, a well‐established proxy measure for socio‐economic status, was calculated by assigning Massachusetts‐specific ADI deciles (1 to 10, higher is more disadvantaged) based on the street address and nine‐digit zip code from the EMR [27]. Home medications at the time of admission included hydroxyurea and home‐based pain regimen.

Hospital day‐level data included pain scores from a numeric rating scale (0 = no pain; 10 = worse pain). The maximum and minimum pain scores were abstracted for each hospital day; our analysis focuses on maximum daily pain scores. Daily medications including opioids and nonopioid pain medications were abstracted. Standard inpatient treatment protocols were not used across all patients; instead, inpatient pain regimens were tailored to the individual patient, based on their standing home regimen and medical history.

For each hospitalization, we calculated the 30‐day unplanned readmission rate for a VOC from the hospital billing data. Readmission rates were restricted to patients who were discharged home after that hospitalization (ie, excluding deaths or those discharged to other facilities) and who were discharged prior to December 1, 2016 (to ensure ≥30 days of follow‐up). VOC hospitalizations from outside hospitals that were documented in our EMR were included when calculating the 30‐day unplanned readmission rate.

### 2.3 Descriptive analysis

Patient and hospitalization characteristics were described for the full sample at the patient and hospitalization level using means, standard deviations (SD), medians, 25th‐75th quartiles (Q1, Q3), frequencies, and percentages. For patient characteristics that could vary over time, characteristics at the first hospitalization were used for the patient‐level description. Data were analyzed using R (3.6.2 for Windows)/R Studio Desktop (1.2.5003) and SAS Software 9.4/SAS Enterprise Guide 7.1 (SAS Institute, Inc Cary, NC) and used a two‐sided alpha of .05.

### 2.4 Identifying clusters of pain trajectories

We used a two‐step approach to identify clusters of VOC hospitalizations with similar pain trajectories: (a) estimate intercepts and slopes of maximum daily pain scores for each hospitalization and (b) estimate finite mixture models using these intercepts and slopes for each hospitalization to identify clusters [14]. Briefly, a linear regression model was fit for each hospitalization with daily maximum pain score as the outcome and hospitalization day as the only covariate. Intercepts and slopes for each hospitalization were output from the linear regression models and included as input for the Gaussian finite mixture model, which allows different covariance structures and different numbers of mixture components (ie, clusters) (see Appendix for detailed description of this analysis and the sensitivity analysis).

To plot the pain trajectories by cluster, predicted values and 95% confidence intervals were estimated using a mixed effects model with random intercepts and slopes for each hospitalization (proc mixed in SAS). Maximum daily pain score was regressed on cluster, hospitalization day, and their interaction, assuming an autoregressive covariance structure (based on Akaike information criteria). Pain trajectories by cluster were plotted from hospitalization day 1 to 7, with 7 representing the 75th percentile of LOS.

When interpreting differences in pain scores, research suggests that 1‐point changes on the 0 to 10 pain scale represent minimally important change, whereas 2‐point changes represent more meaningful decreases in pain [28].

### 2.5 Analysis of factors associated with clusters of pain trajectories

We identified patient and hospitalization characteristics that were associated with clusters of pain trajectories, while accounting for the correlation of hospitalizations within patients using generalized estimating equations (proc GEE in SAS) with a multinomial distribution (glogit link) and an independent covariance structure [29]. The following covariates were included in the multivariable model based on a priori hypothesized relationships with pain experience: gender, affective disorder, number of SCD complications, home use of hydroxyurea, and genotype (Hb SS vs all others).

We hypothesized that daily use of opioids would be associated with clusters of pain trajectories. However, we were unable to assess for this relationship because nearly all hospitalization days (98%) involved opioid use and daily morphine equivalent dose (MED) was not available for all patients during the study period.

### 2.6 Analysis of relationship between clusters of pain trajectories and 30‐day readmission

This analysis was restricted to patients who were established at Tufts MC because they would be expected to be readmitted at Tufts MC (see Table A2 for characteristics of established patients). Using GEE with a binomial distribution and logit link (with an autoregressive covariance structure based on quasi‐likelihood criteria) [30] to account for the correlation of hospitalizations within patients, we tested the association between clusters of pain trajectories and 30‐day readmissions. The multivariable model was fit adjusting for gender, age, affective disorder, number of SCD complications, home use of hydroxyurea, and genotype (Hb SS vs all others).

### 2.7 Analysis of LOS

We hypothesized that longer LOS was associated with clusters with slower decrease in pain scores. Given this collinearity, both LOS and cluster were not included in the primary multivariable analysis of 30‐day readmissions. First, we assessed the univariate relationship between LOS and clusters of pain trajectories. Next, we did a sensitivity analysis assessing the relationship between LOS and 30‐day readmissions. This included the univariate relationship between LOS and 30‐day readmissions, as well as adding LOS to the primary multivariable model for 30‐day readmissions. In addition, for 30‐day readmissions, we calculated the concordance statistic (c‐statistic) based on the area under the receiver operator characteristic curve for the primary multivariable model and the models from the sensitivity analysis to determine which model better discriminated among those who had 30‐day readmissions. The c‐statistic ranges from 0.5 to 1, with higher scores indicating better predictive accuracy of the model.

## 3 RESULTS

### 3.1 Sample

Initially, 568 hospitalizations among 90 patients with SCD were identified as potentially eligible. After applying inclusion/exclusion criteria, the analytic cohort included 350 hospitalizations for VOC among 59 patients (Figure 1). The median number of hospitalizations per patient over the study period was 3 (Q1 = 1, Q3 = 7); 19 patients had only one hospitalization, whereas one patient had 33 hospitalizations.

Among the 59 patients, 55.9% were female, 76.3% were black, and Hb SS was the most common genotype (Table 1). At their first hospitalization in the study window, the median age at admission was 26 years (Q1 = 21, Q3 = 29), 35.6% had a documented affective disorder, the mean number of SCD complications was 2.7 (SD = 1.5), and 57.6% were established patients. At the hospitalization level, median LOS was 7 days (Q1 = 5, Q3 = 9).

**TABLE 1:** Patient and hospitalization characteristics

	Patient‐level, n = 59	Hospitalization‐level, n = 350
Patient characteristics^a^		
Age in years at admission, median (Q1, Q3)	26 (21, 29)	24 (20, 30)
Female, n (%)	33 (55.9%)	226 (64.6%)
Race/ethnicity, n (%)		
Black/African American	45 (76.3%)	251 (71.7%)
Hispanic	12 (20.3%)	95 (27.1%)
Other	2 (3.4%)	4 (1.1%)
Insurance, n (%)		
Any Medicare	16 (27.1%)	122 (34.9%)
Medicaid/No Medicare	37 (62.7%)	202 (57.7%)
Private only	6 (10.2%)	26 (7.4%)
SCD genotype, n (%)		
Hb SS	36 (61.0%)	245 (70.0%)
Hb SC	13 (22.0%)	79 (22.6%)
Other^b^	10 (17.0%)	26 (7.4%)
Number of SCD complications, mean (SD)	2.7 (1.5)	3.5 (1.3)
Affective disorder, n (%)	21 (35.6%)	178 (50.9%)
Prescribed hydroxyurea, n (%)^c^	35 (60.3%)	275 (79.3%)
ADI score, median (Q1, Q3)^d^	6 (6, 8)	7 (6, 9)
Established patient, n (%)	34 (57.6%)	293 (83.7%)
Hospitalization characteristics		
LOS in days, median (Q1, Q3)	n/a	7 (5, 9)
Admission pain score, median (Q1, Q3)	n/a	9 (8, 10)
Discharge pain score, median (Q1, Q3)	n/a	5 (4, 6)

^a^ For time‐varying patient characteristics, only the first hospitalization was included.

^b^ Other includes Hb Sβ^+^ thalassemia, Hb Sβ^0^ thalassemia, unknown genotype (two patients and three hospitalizations are unknown).

^c^ One patient and three hospitalizations did not have information in the EMR about their home medication regimen.

^d^ Seven patients and 38 hospitalizations were missing ADI score.

Abbreviations: ADI, Area Deprivation Index; EMR, electronic medical record; Hb, hemoglobin; LOS, length of stay; SCD, sickle cell disease.

### 3.2 Clusters of pain trajectories

**FIGURE 2:**
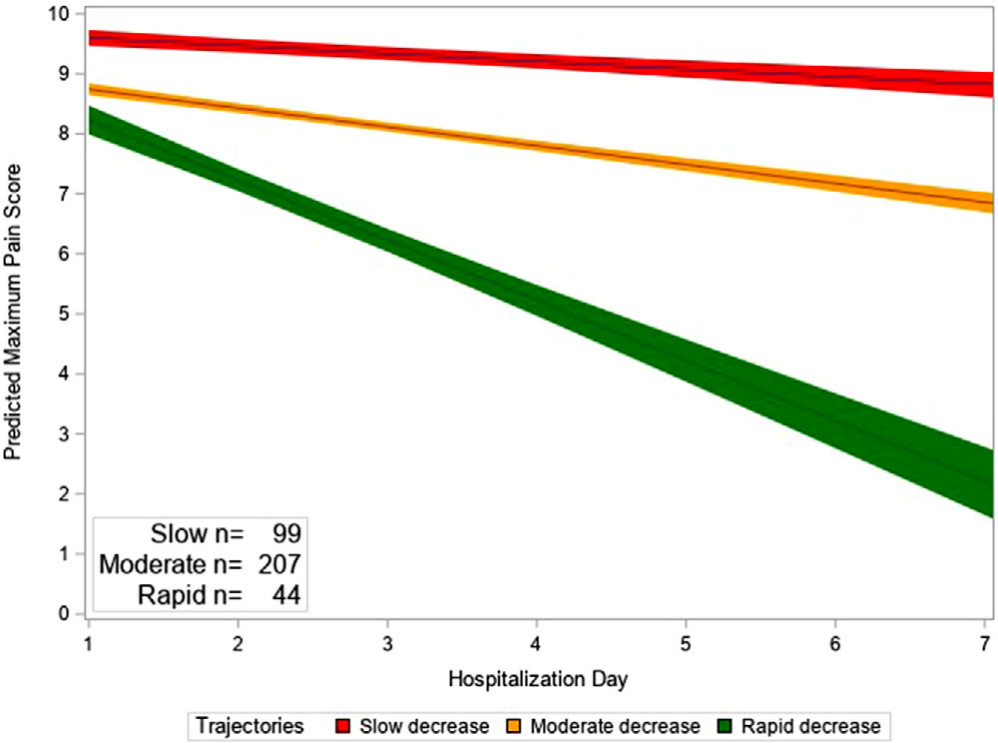
Predicted maximum daily pain score based on trajectories *Note*. Bands represent 95% confidence limits.

Across all hospitalizations, the mean intercept was 9.03 (SD = 1.06) and the mean slope was –0.43 (SD = 0.58). Three distinct clusters of hospitalizations with similar pain trajectories were identified using finite mixture models and were described based on the steepness of the pain slope over the course of hospitalization: (a) slow decrease (n = 99, intercept = 9.58, slope = –0.14); (2) moderate decrease (n = 207, intercept = 8.89, slope = –0.36); and rapid decrease in pain scores (n = 44, intercept = 8.60, slope = –1.13) (Figure 2). Hospitalizations with slow decrease in pain scores (ie, shallow slope) had the highest initial pain score (ie, intercept), followed by hospitalizations with moderate decrease in pain scores, and then hospitalizations with rapid decrease in pain scores. Among the 40 patients who had more than one hospitalization, only eight had hospitalizations that were always classified in the same cluster; the remaining 32 patients had hospitalizations in at least two different clusters. See Table A3 for results of sensitivity analysis.

### 3.3 Factors associated with clusters of pain trajectories

Patient and hospital characteristics were described by clusters of pain trajectories (Table 2), and univariate and multivariable analyses identified factors associated with the clusters (Table 3). Univariate analysis found that hospitalizations among patients who were female (odds ratio [OR] = 2.56; 95% confidence interval [CI], 1.10‐5.98) and were prescribed hydroxyurea (OR = 2.27; 95% CI, 1.0‐5.11) had higher odds of being in the cluster with moderate decrease in pain scores than the cluster with rapid decrease. Hospitalizations among patients with more SCD complications (OR = 1.88; 95% CI, 1.25‐2.84) and affective disorders (OR = 4.05; 95% CI, 1.46‐11.20) had higher odds of being in the cluster with slow decrease in pain scores than the cluster with rapid decrease. In multivariable analysis, hospitalizations among female patients (OR = 2.61; 95% CI, 1.32‐5.14) had higher odds of being in the cluster with moderate decrease in pain scores than the cluster with rapid decrease. Hospitalizations among patients with more SCD complications (OR = 1.56; 95% CI, 1.04‐2.34) or with an affective disorder (OR = 3.27; 95% CI, 1.11‐9.65) had higher odds of being in the cluster with slow decrease in pain scores than the cluster with rapid decrease.

**TABLE 2:** Patient and hospitalization characteristics by clusters of pain trajectories (n = 350)

	Slow decrease in pain (n = 99)	Moderate decrease in pain (n = 207)	Rapid decrease in pain (n = 44)
Patient characteristics			
Age in years at admission, median (Q1, Q3)	29 (21, 32)	23 (19, 29)	24 (21, 27)
Female, n (%)	60 (60.6%)	145 (70.1%)	21 (47.7%)
Race/ethnicity, n (%)			
Black/African American	76 (76.8%)	143 (69.1%)	32 (72.7%)
Hispanic	23 (23.2%)	61 (29.5%)	11 (25.0%)
Other	0 (0.0%)	3 (1.5%)	1 (2.3%)
Insurance, n (%)			
Any Medicare	47 (47.5%)	60 (29.0%)	15 (34.1%)
Medicaid/No Medicare	49 (49.5%)	129 (62.3%)	24 (54.6%)
Private only	3 (3.0%)	18 (8.7%)	5 (11.4%)
SCD genotype, n (%)			
Hb SS	76 (76.8%)	141 (68.1%)	28 (63.6%)
Hb SC	19 (19.2%)	51 (24.6%)	9 (20.5%)
Other^a^	4 (4.0%)	15 (7.3%)	7 (15.9%)
Number of SCD complications, median (Q1, Q3)	4 (3, 5)	3 (3, 4)	3 (2, 3)
Affective disorder, n (%)^b^	67 (67.7%)	96 (46.4%)	15 (34.1%)
Prescribed hydroxyurea, n (%)^c^	79 (80.6%)	167 (81.5%)	29 (65.9%)
ADI score, median (Q1, Q3)^d^	7 (6, 9)	6 (6, 8)	7 (6, 9)
Established patient, n (%)	79 (79.8%)	183 (88.4%)	31 (70.5%)
Hospitalization characteristics			
LOS in days, median (Q1, Q3)	9 (7, 11)	7 (5, 8)	4 (3, 5)
Admission pain score, median (Q1, Q3)	9 (8, 10)	8 (8, 10)	8 (7, 10)
Discharge pain score, median (Q1, Q3)	6 (5, 8)	5 (4, 6)	4 (1.5, 4)

^a^ For time‐varying patient characteristics, only the first hospitalization was included.

^b^ Other includes Hb Sβ^+^ thalassemia, Hb Sβ^0^ thalassemia, unknown genotype (two patients and three hospitalizations are unknown).

^c^ Three hospitalizations did not have information in the EMR about their home medication regimen.

^d^ Thirty‐eight hospitalizations were missing ADI score.

Abbreviations: ADI, Area Deprivation Index; EMR, electronic medical record; Hb, hemoglobin; LOS, length of stay; SCD, sickle cell disease.

**TABLE 3:** Multinomial logistic regression results of factors associated with clusters of pain trajectories (n = 350)

	Univariate, OR (95% CI)		Multivariable, OR (95% CI)	
	Moderate versus rapid decrease in pain	Slow versus rapid decrease in pain	Moderate versus rapid decrease in pain	Slow versus rapid decrease in pain
Female	**2.56 (1.10‐5.98)**	1.68 (0.47‐6.06)	**2.61 (1.32‐5.14)**	1.23 (0.32‐4.71)
Hb SS genotype	1.22 (0.54‐2.78)	1.89 (0.54‐6.63)	0.85 (0.36‐2.03)	1.62 (0.31‐8.48)
Number of SCD complications	1.17 (0.84‐1.62)	**1.88 (1.25‐2.84)**	1.03 (0.70‐1.52)	**1.56 (1.04‐2.34)**
Affective disorder	1.67 (0.84‐3.32)	**4.05 (1.46‐11.20)**	1.45 (0.64‐3.31)	**3.27 (1.11‐9.65)**
Prescribed hydroxyurea	**2.27 (1.01‐5.11)**	2.15 (0.84‐5.54)	2.27 (0.94‐5.45)	1.27 (0.44‐3.67)

*Note*. Bolding indicates *P* < .05.

**TABLE 4:** Logistic regression results for 30‐day readmissions (n = 280)

	Univariate	Multivariable
	OR (95% CI)	OR (95% CI)
Clusters of pain trajectories		
Rapid versus slow decrease in pain	0.58 (0.23‐1.43)	0.68 (0.27‐1.71)
Moderate versus slow decrease in pain	**0.54 (0.31‐0.96)**	0.60 (0.33‐1.06)
Age in years at admission	0.96 (0.92‐1.01)	**0.92 (0.88‐0.96)**
Female	0.68 (0.29‐1.58)	0.65 (0.26‐1.60)
Hb SS genotype	2.00 (0.61‐6.59)	1.44 (0.61‐3.39)
Number of SCD complications	**1.71 (1.21‐2.42)**	**1.85 (1.19‐2.89)**
Affective disorder	1.74 (0.89‐3.39)	1.55 (0.80‐3.02)
Prescribed hydroxyurea	1.33 (0.66‐2.68)	0.71 (0.37‐1.37)

*Note*. Bolding indicates *P* < .05.

### 3.4 Relationship between clusters of pain trajectories and 30‐day readmission rate

Among the 280 hospitalizations for established patients, 122 (43.6%) had a 30‐day readmission. Univariate analysis found that hospitalizations in the cluster with moderate decrease in pain scores (OR = 0.54; 95% CI, 0.31‐0.96) had lower odds of 30‐day readmissions than hospitalizations in the cluster with slow decrease; and hospitalizations for patients with more SCD complications (OR = 1.71; 95% CI, 1.21‐2.42) had higher odds of readmission (Table 4). In multivariable analysis, hospitalizations among older patients had lower odds of 30‐day readmission (OR = 0.92; 95% CI, 0.88‐0.96), whereas hospitalizations for patients with more SCD complications had higher odds of 30‐day readmission (OR = 1.85; 95% CI, 1.19‐2.89).

### 3.5 Analysis of LOS

Hospitalizations with longer LOS had higher odds of being in the cluster with moderate decrease (OR = 1.76; 95% CI, 1.41‐2.18) and the cluster with slow decrease (OR = 2.25; 95% CI, 1.73‐2.92) than the cluster with rapid decrease in pain scores. LOS was not associated with 30‐day readmission in univariate(OR = 0.97; 95% CI, 0.89‐1.06) or multivariable (OR = 0.94; 95% CI, 0.85‐1.04) analysis. Clusters of pain trajectories (0.56; SE = 0.03) had a higher c‐statistic for the univariate relationship with 30‐day readmission than LOS (0.50; SE = 0.03); the c‐statistic for the primary multivariable model for 30‐day readmission was 0.75 (SE = 0.03), which did not change after adding LOS (c‐statistic = 0.75; SD = 0.3).

## 4 DISCUSSION

Based on 350 VOC hospitalizations among 59 patients with SCD, we identified three clusters of hospitalizations with similar pain trajectories (rapid, moderate, and slow decrease); the cluster with moderate decrease in pain scores was the most common. Hospitalizations among females, patients with more SCD complications, or patients with affective disorders had higher odds of being in the cluster with worse pain trajectories (ie, slow or moderate decrease in pain scores). Although hospitalizations in the cluster with moderate decrease in pain scores had lower odds of 30‐day readmissions than the cluster with slow decrease, this was no longer significant in the multivariable model. Younger age and more SCD complications were associated with higher odds of 30‐day readmissions.

This analysis provides an advancement over prior studies of pain trends during VOC hospitalizations, which were primarily descriptive [7, 8, 9]. We used a data‐driven approach to uncover clusters of hospitalizations with similar pain trajectories, highlighting variability in pain experiences. As previously observed, we found that pain tended to decrease from admission to discharge, although at different rates across the clusters. Our approach also allowed us to test whether patient and clinical characteristics were associated with pain trajectory clusters. By its nature, the use of finite mixture models to identify clusters of hospitalizations with similar pain trajectories is exploratory, so there is no correct number of clusters. Although our primary analysis identified three clusters, our sensitivity analysis found only two, indicating that the number of clusters is sensitive to the criteria we used to fit the model. Differences in pain scores across the three clusters, as well as differences in patient and clinical characteristics across the clusters, provide initial justification for the use of three clusters. Additional research that includes more patients from multiple hospitals should be conducted to further validate these findings. The number of clusters of hospitalizations with similar pain trajectories could also be determined by how the results will be used, and clinical criteria should also be incorporated.

Although a widely used and validated measure of SCD severity does not currently exist, frequent VOCs and more SCD‐related complications likely represent higher disease severity [15, 16, 17]. Our results confirmed that hospitalizations for patients with more SCD complications had slower decreases in pain scores and more SCD complications were associated with 30‐day readmissions. In fact, the relationship between clusters of pain trajectories and 30‐day readmissions likely attenuated from statistically significant to nonsignificant after adjustment for SCD complications. LOS is closely related to pain trajectories and disease severity, as patients with rapid decreases in pain scores have shorter LOS. Given this relationship, we did not include LOS in our primary analysis. However, sensitivity analyses found that LOS was not associated with 30‐day readmissions and that pain trajectory clusters alone were better at predicting 30‐day readmissions than LOS alone. Hospital discharge often depends on a return to baseline pain level, rather than complete resolution. Unfortunately, we were unable to collect information about the baseline pain experience outside the hospital.

Aside from SCD complications, we found that affective disorders and female gender were associated with less rapid resolution of pain trajectories. The relationship between affective disorders and the pain experience is complicated and bidirectional; pain may lead to an increase in anxiety or depression, whereas anxiety or depression may lead to differences in the pain experience [18, 19]. Prior research shows that males have more severe SCD and lower life expectancy than females [31, 32]. However, other studies found no differences in the pain experience by gender or possible differences related to the menstrual cycle [33, 34]. A possible explanation is that females experience more anxiety and depression, which could result in different pain experiences, but our results adjusted for affective disorder [18, 35].

Age was the only factor besides SCD complications that was associated with 30‐day readmissions. Older age was actually associated with lower 30‐day readmission rates, which has been observed in other studies of SCD [1]. Interestingly, the univariate relationship between age and 30‐day readmissions was not significant, but became significant after adjustment for SCD complications. Given life expectancy in the 40s or 50s, we hypothesize that patients with more severe SCD die sooner, so patients who survive to older adulthood have less severe disease and lower healthcare utilization [32, 36, 37]. Of note, this study only included patients ≥18 years old so this age‐based relationship may be different for pediatric patients.

Results from this study could be used in clinical care to help identify vulnerable subgroups of patients with SCD, such as those with more SCD complications or affective disorders, who are at risk for worse pain trajectories. Ideally, treatment with hydroxyurea, blood transfusions, and more recently approved therapies (eg, L‐glutamine, crizanlizumab, and voxelotor) would reduce the frequency and severity of VOC and associated SCD complications [38]. However, for patients who continue to experience SCD complications, we can use our results to improve care. For example, targeted follow‐up care or treatment could be based on patient‐reported tools for assessing SCD complications, such as the ASCQ‐Me SCD Medical History Checklist [17]. Similarly, patients should be screened for affective disorders and referred to appropriate treatment.

We acknowledge this study's limitations. Although the number of patients was not large, we included 350 hospitalizations and used methods to account for multiple hospitalizations per patient. This study was conducted at a single medical center, which may reduce generalizability based on patient characteristics or practice patterns. Patients’ pain treatment protocols, including their daily MED, could have influenced their pain trajectories, but daily MED was not available on all patients during the study period. Exclusions of hospitalizations based on LOS may also affect generalizability as these patients may reflect patients with more severe disease based on the number of SCD complications. Readmissions to outside hospitalizations could have been missed, but this is unlikely given that we restricted our analysis to established patients, we included outside hospitalizations that were documented in our EMR, and our readmission rate was comparable to other studies [39, 40].

In conclusion, we identified three unique clusters of pain trajectories among VOC hospitalizations using a novel, data‐driven approach. SCD complications were associated with slower decreases in pain scores as well as increased 30‐day readmissions. These results highlight the variability in pain trajectories among patients with SCD experiencing VOC and provide a novel approach for identifying subgroups of patients who are most vulnerable and could benefit from interventions, such as more intensive follow‐up.

## FUNDING INFORMATION

National Institute on Minority Health and Health Disparities (R21 MD011455‐01) and National Center for Advancing Translational Sciences (1KL2TR002545).

## AUTHOR CONTRIBUTIONS

AMR conceptualized the study; acquired the data; analyzed the data and created the figures and tables; acquired funding support; and wrote and edited the paper. KSE contributed to the study design; helped acquire the data; and reviewed and edited the paper. NSS helped acquire the data and reviewed and edited the paper. SKP conceptualized the study; helped acquire the data; helped acquire funding support; and reviewed and edited the paper.

## CONFLICT OF INTEREST

The authors declare no conflict of interest.

## APPENDIX 1 APPENDIX

### METHODS

#### SCD complications

The 13 SCD complications included stroke; osteonecrosis/avascular necrosis; cholecystectomy or cholecystitis; hyperhemolysis/hyperhemolytic syndrome; acute renal failure or chronic kidney disease; pulmonary hypertension or acute chest syndrome; priapism; retinopathy; silent infarct lesion on MRI; sickle hepatopathy; splenectomy, hypersplenism, and splenic sequestration; thrombosis; and leg or foot ulcer/sores. Each complication type was only recorded once (eg, yes/no acute chest syndrome). Thus, the number of SCD complications reflects the different types of complications experienced by the patient, not the number of episodes of a given complication. Two hematologists reviewed each patient's SCD complications after extraction by trained study staff.

**TABLE A1:** Hospitalization characteristics by length of stay (n = 449)

	<3 days, n = 39	3‐14 days, n = 350	>14 days, n = 60
Patient characteristics^a^			
Age in years at admission, median (Q1, Q3)	26 (23, 30)	24 (20, 30)	29 (23, 34.5)
Female, n (%)	16 (41.0%)	226 (64.6%)	47 (78.3%)
Race/ethnicity, n (%)			
Black/African American	30 (76.9%)	251 (71.7%)	41 (68.3%)
Hispanic	9 (23.1%)	95 (27.1%)	19 (31.7%)
Other	0 (0.0%)	4 (1.1%)	0 (0.0%)
Insurance, n (%)			
Any Medicare	17 (43.6%)	122 (34.9%)	25 (41.7%)
Medicaid/No Medicare	22 (56.4%)	202 (57.7%)	31 (51.7%)
Private only	0 (0.0%)	26 (7.4%)	4 (6.7%)
SCD genotype, n (%)			
Hb SS	22 (56.4%)	245 (70.0%)	47 (78.3%)
Hb SC	14 (35.9%)	79 (22.6%)	9 (15.0%)
Other^b^	3 (7.7%)	26 (7.4%)	4 (6.7%)
Number of SCD complications, mean (SD)	3 (1.5)	3.5 (1.3)	4 (3, 5)
Affective disorder, n (%)	13 (33.3%)	178 (50.9%)	38 (63.3%)
Prescribed hydroxyurea, n (%)^c^	29 (76.3%)	275 (79.3%)	47 (78.3%)
ADI score, median (Q1, Q3)^d^	7 (6, 8)	7 (6, 9)	6 (6, 9)
Established patient, n (%)	23 (59.0%)	293 (83.7%)	51 (85.0%)
Hospitalization characteristics			
LOS in days, median (Q1, Q3)	2 (2, 2)	7 (5, 9)	19 (16.5, 24)
Admission pain score, median (Q1, Q3)	8 (8, 9)	9 (8, 10)	9 (8, 10)
Discharge pain score, median (Q1, Q3)	4 (3, 6)	5 (4, 6)	5 (4, 7)
Utilization outcome			
30‐day readmission, n (%)	20 (51.3%)	139 (41.4%)	25 (43.1%)

^a^ For time‐varying patient characteristics, only the first hospitalization was included.

^b^ Other includes Hb Sβ^+^ thalassemia, Hb Sβ^0^ thalassemia, unknown genotype (three hospitalizations are unknown).

^c^ Four hospitalizations did not have information in the EMR about their home medication regimen.

^d^ Forty‐three hospitalizations were missing ADI score.

^e^ Sixteen hospitalizations were excluded from 30‐day readmission due to not being discharged home or being discharged within 30 days of study end date.

Abbreviations: ADI, Area Deprivation Index; EMR, electronic medical record; Hb, hemoglobin; LOS, length of stay; SCD, sickle cell disease.

**TABLE A2:** Patient and hospitalization characteristics by established patients, restricted to analytic cohort (n = 350)

	Patient‐level		Hospitalization‐level	
	Not established, n = 25	Established, n = 34	Not established, n = 57	Established, n = 293
Patient characteristics^a^				
Age in years at admission, median (Q1, Q3)	26 (23, 28)	25 (20, 32)	24 (23, 28)	24 (20, 31)
Female, n (%)	9 (36.0%)	24 (70.6%)	18 (31.6%)	208 (71.0%)
Race/ethnicity, n (%)				
Black/African American	20 (80.0%)	25 (73.5%)	43 (75.4%)	208 (71.0%)
Hispanic	3 (12.0%)	9 (26.5%)	11 (19.3%)	84 (28.7%)
Other	2 (8.0%)	0 (0.0%)	3 (5.3%)	1 (0.3%)
Insurance, n (%)				
Any Medicare	5 (20.0%)	11 (32.4%)	21 (36.8%)	101 (34.5%)
Medicaid/No Medicare	16 (64.0%)	21 (61.8%)	31 (54.4%)	171 (58.4%)
Private only	4 (16.0%)	2 (5.9%)	5 (8.8%)	21 (7.2%)
SCD genotype, n (%)				
Hb SS	11 (44.0%)	25 (73.5%)	31 (54.4%)	214 (73.0%)
Hb SC	6 (24.0%)	7 (20.6%)	9 (15.8%)	70 (23.9%)
Other^b^	8 (32.0%)	2 (5.9%)	17 (29.8%)	9 (3.1%)
Number of SCD complications, mean (SD)	3 (1, 3)	3 (2, 3)	3 (2, 4)	3 (3, 4)
Affective disorder, n (%)	3 (12.0%)	18 (52.9%)	11 (19.3%)	167 (57.0%)
Prescribed hydroxyurea, n (%)^c^	12 (50.0%)	23 (67.7%)	38 (69.1%)	237 (81.2%)
ADI score, median (Q1, Q3)^d^	6 (6, 9)	7 (6, 8)	7 (4.5, 9)	7 (6, 9)
Hospitalization characteristics				
LOS in days, median (Q1, Q3)	n/a	n/a	6 (4, 9)	7 (5, 9)
Admission pain score, median (Q1, Q3)	n/a	n/a	9 (8, 10)	9 (8, 10)
Discharge pain score, median (Q1, Q3)	n/a	n/a	5 (4, 6)	5 (4, 6)
Utilization outcome				
30‐day readmission, n (%)	n/a	n/a	17 (30.4%)	122 (43.6%)

^a^ For time‐varying patient characteristics, only the first hospitalization was included.

^b^ Other includes Hb Sβ^+^ thalassemia, Hb Sβ^0^ thalassemia, unknown genotype (two patients and three hospitalizations are unknown).

^c^ One patient and three hospitalizations did not have information in the EMR about their home medication regimen.

^d^ Seven patients and 38 hospitalizations were missing ADI score.

^e^ Fourteen hospitalizations were excluded from 30‐day readmission due to not being discharged home or being discharged within 30 days of study end date.

Abbreviations: ADI, Area Deprivation Index; EMR, electronic medical record; Hb, hemoglobin; LOS, length of stay; SCD, sickle cell disease.

#### Identifying clusters of pain trajectories

We used a two‐step approach to identify clusters of VOC hospitalizations with similar pain trajectories: (a) estimate intercepts and slopes of maximum daily pain scores for each hospitalization and (b) estimate finite mixture models using these intercepts and slopes for each hospitalization to identify clusters [14]. For each individual hospitalization, we fit a linear regression model with daily maximum pain score as the outcome and hospitalization day as the only covariate (using the lm function in R). Visual inspection of plots of observed daily pain scores over the course of each hospitalization justified the use of linear regression. Intercepts and slopes for each hospitalization were output from the linear regression models and included as input for the finite mixture model (using mclust package in R). Gaussian finite mixture models were fit via the expectation‐maximization (EM) algorithm, allowing for different covariance structures and different numbers of mixture components (ie, clusters). Clusters are ellipsoidal, centered at the mean, with volume, shape, and orientation determined by the covariance matrix. In the multivariate setting, the volume, shape, and orientation of the covariance can be equal or unequal across clusters. The number of clusters was based on the following criteria: Bayesian information criteria (BIC) and ≥10% of sample in each cluster. BIC was also used to determine the covariance structure. As a sensitivity analysis, the integrated complete‐data likelihood (ICL) was used instead of the BIC to determine the number of clusters. Both BIC and ICL are based on a penalized form of the log‐likelihood and provide a method for comparing models [14].

### RESULTS

#### Clusters of pain trajectories

The covariance was unequal across clusters for volume, shape, and orientation. A sensitivity analysis using ICL rather than BIC identified two clusters of hospitalizations with similar pain trajectories (Table A3).

**TABLE A3:** Description of GMM models, primary and sensitivity analyses

	Primary GMM based on BIC	Sensitivity analysis of GMM based on ICL
Number of clusters	3	2
Sample size of clusters	99; 207; 44	303; 47
Intercepts of clusters	9.58; 8.89; 8.60	9.12; 8.54
Slopes of clusters	−0.14; −0.36; −1.13	−0.29; −1.15
Covariance	Unequal volume; unequal shape; unequal orientation	Unequal volume; equal shape; unequal orientation

Abbreviations: BIC, Bayes information criteria; ICL, integrated complete‐data likelihood.
